# Expression and Activity of the Transcription Factor CCAAT/Enhancer-Binding Protein β (C/EBPβ) Is Regulated by Specific Pulse-Modulated Radio Frequencies in Oligodendroglial Cells

**DOI:** 10.3390/ijms241311131

**Published:** 2023-07-05

**Authors:** Bing Huang, Weihao Zhao, Xue Cai, Yumin Zhu, Yingxian Lu, Junli Zhao, Nan Xiang, Xiaofei Wang, Hu Deng, Xiaping Tang, Lingyu Liu, Yanyu Zhao, Yigong Shi

**Affiliations:** 1 Zhejiang Provincial Laboratory of Life Sciences and Biomedicine, Xihu District, Hangzhou 310024, China; 2 Brain Function and Disease Laboratory, Department of Pharmacology, Shantou University Medical College, Xin-Ling Road #22, Shantou 515041, China; 3 Institute of Biology, Westlake Institute for Advanced Study, Key Laboratory of Structural Biology of Zhejiang Province, School of Life Sciences, Westlake University, Hangzhou 310024, China; 4 Program in Computational Biology and Bioinformatics, Department of Molecular Biophysics and Biochemistry, Yale University, New Haven, CT 06520, USA; 5 iMarker Lab, Westlake Laboratory of Life Sciences and Biomedicine, Key Laboratory of Structural Biology of Zhejiang Province, School of Life Sciences, Westlake University, Hangzhou 310024, China; 6 MOE Key Laboratory of Population Health Across Life Cycle, Anhui Provincial Key Laboratory of Population Health and Aristogenics, Department of Maternal & Child and Adolescent Health, School of Public Health, Anhui Medical University, Hefei 230032, China; 7 Beijing Advanced Innovation Center for Structural Biology & Frontier Research Center for Biological Structure, Tsinghua-Peking Joint Center for Life Sciences, School of Life Sciences, Tsinghua University, Beijing 100084, China

**Keywords:** radio frequency (RF), 2.4 GHz, pulse modulated, oligodendrocyte, CCAAT/enhancer-binding protein β (C/EBPβ)

## Abstract

The rapid growth of wireless electronic devices has raised concerns about the harmful effects of leaked electromagnetic radiation (EMR) on human health. Even though numerous studies have been carried out to explore the biological effects of EMR, no clear conclusions have been drawn about the effect of radio frequency (RF) EMR on oligodendrocytes. To this end, we exposed oligodendroglia and three other types of brain cells to 2.4 GHz EMR for 6 or 48 h at an average input power of 1 W in either a continuous wave (CW-RF) or a pulse-modulated wave (PW-RF, 50 Hz pulse frequency, 1/3 duty cycle) pattern. RNA sequencing, RT-qPCR, and Western blot were used to examine the expression of C/EBPβ and its related genes. Multiple reaction monitoring (MRM) was used to examine the levels of expression of C/EBPβ-interacting proteins. Our results showed that PW-RF EMR significantly increased the mRNA level of C/EBPβ in oligodendroglia but not in other types of cells. In addition, the expression of three isoforms and several interacting proteins and targeted genes of C/EBPβ were markedly changed after 6-h PW-RF but not CW-RF. Our results indicated that RF EMR regulated the expression and functions of C/EBPβ in a waveform- and cell-type-dependent manner.

## 1. Introduction

The growing number of modern electronic devices that work with distinct electromagnetic wavelengths has raised concerns about the hazards of EMR to human health [1]. RF EMR (ranging from 3 kHz to 300 GHz) covers the wavelengths of mobile phones, Bluetooth, and Wi-Fi devices that use frequencies of around 2.4 GHz. The potential health risks of these wireless devices are still unclear, leading to challenges for medicine and society. Extensive research has used a variety of systems to study the effects of EMR at the cellular, animal, and epidemiological levels, whereas the association between EMR and health is still highly controversial.

In the central nervous system (CNS), neurons are the most characteristic cells and are mainly responsible for information transduction. The speed and efficiency of action potentials in neurons rely on myelin, a sheath structure around axons that is generated by oligodendrocytes. Along with their axon insulation capability, oligodendrocytes also provide neurons with metabolic support [2] and induce sodium channel clustering along the axons at the node of Ranvier, which is an important prerequisite for saltatory nerve conduction [3]. Besides their essential function in neurodegenerative diseases such as Amyotrophic Lateral Sclerosis (ALS) [4], oligodendrocytes are reported to be modulators in depression disorders [5], sleep and wakefulness [6], and glioblastomas [7]. However, nothing is known about the effects of RF EMR on the expression or function of genes in oligodendrocytes.

C/EBPβ belongs to a transcription factor family (C/EBPα–ζ), the members of which share a basic leucine zipper domain in the C-terminal for DNA binding and dimerization [8]. Homodimeric and heterodimeric interactions occur not only between isoforms but also between different family members [9]. C/EBPβ mRNA yields three different protein isoforms generated from a single exon gene via leaky ribosome scanning: the full-length liver-enriched activator protein (38-kDa LAP1), the 34-kDa liver-enriched activator protein (LAP2), and the 21-kDa liver-enriched inhibitory protein (LIP) [10]. C/EBPβ is reported to regulate extensive physiological activities, including autophagy [11], the differentiation of myeloid lineages [12], inflammation [13], synaptic plasticity [14], and the etiology of glioblastoma [15]. Whether and how RF EMR might influence C/EBPβ expression and function in oligodendrocytes is still unknown.

Here, we exposed four types of brain cells to 2.4 GHz EMR in continuous or pulse-modulated waveforms under an average input power of 1 W. Our results showed that in oligodendroglia, but not in neuronal cells, microglial cells, or primary astrocytes, the transcription of C/EBPβ was significantly upregulated after 6 h of exposure to PW-RF but not CW-RF. Consistently, the expression of C/EBPβ was particularly increased in oligodendroglial cells after 6-h PW-RF exposure. Furthermore, 6-h PW-RF exposure specifically increased the expression of C/EBPβ-interacting proteins and the transcriptional activity of C/EBPβ. Our results indicated that the expression and function of C/EBPβ in oligodendroglial cells were specifically regulated by pulse-modulated EMR.

## 2. Results

### 2.1. Exposure to 2.4 GHz EMR Distinctly Regulated the Transcription and Expression of C/EBPβ in Oligodendroglia, Neuronal Cells, Microglial Cells, and Astrocytes

**Figure 1:**
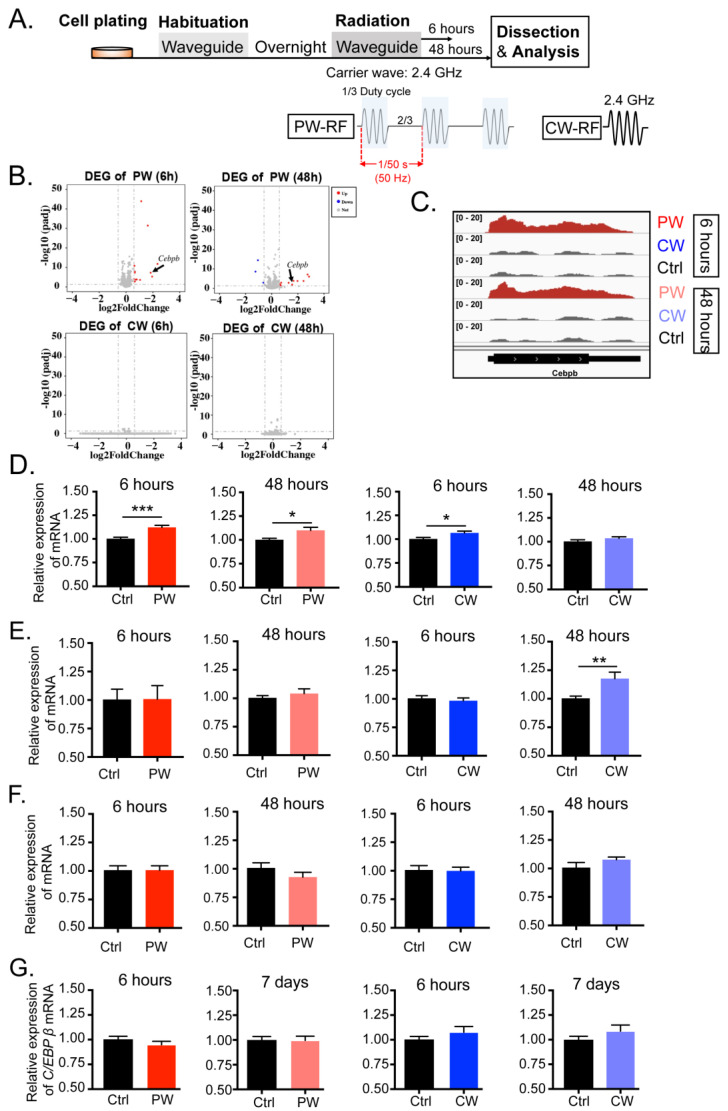
Transcription of C/EBPβ was distinctly altered after 2.4 GHz EMR in oligodendroglial cells, neuronal cells, microglial cells, and astrocytes. (**A**) Experimental procedures. Cells were plated in 35 mm Petri dishes and transferred to the waveguides in designated positions to habituate to the environment overnight. Radiation signals were then turned on to stimulate each group of cell samples for 6 h and 48 h, respectively, after which, cells were dissected to conduct total RNA sequencing or RT-qPCR analysis. PW-RF groups were stimulated by a 2.4 GHz pulse-modulated signal (50 Hz pulse frequency, 1/3 duty cycle), and the CW-RF groups were stimulated by a 2.4 GHz continuous signal. The control (Ctrl) groups did not receive any EMR signals. (**B**) Volcano plot of DEGs of both the PW-RF (**top**) and CW-RF groups (**bottom**) versus their corresponding Ctrl groups (n = 5 per group). Up-regulated DEGs (FDR < 0.05, log_2_ fold change > 0.585) are colored red, and down-regulated DEGs (FDR < 0.05, log_2_ fold change < −0.585) are colored blue. (**C**) IGV image showing enhanced read counts of C/EBPβ after both 6 h and 48 h of PW-RF exposure, corresponding to the results shown in panel (**B**). (**D**–**G**) Relative C/EBPβ mRNA level in oligodendroglial cells (n = 19–20 per group), neuronal cells (n = 15 per group), microglial cells (n = 9–10 per group), and astrocytes (n = 5–10 per group). * *p* < 0.05, ** *p* < 0.01, *** *p* < 0.001 vs. Ctrl.

In order to investigate how 2.4 GHz EMR affects oligodendrocytes, we first examined the transcriptome of a permanent oligodendroglial cell line, OLN-93, after 6 and 48 h of exposure to 2.4 GHz EMR in the waveguide (Figure 1A). Different EMR stimulations were simultaneously applied at different waveguides. For the CW-RF group, the waveform was set as a sinusoidal EMR signal of 2.4 GHz. For the PW-RF group, a sinusoidal signal of the frequency of 2.4 GHz was applied for 6.67 ms, followed by no EMR signal for 13.33 ms (corresponding to the duty cycle of a pulse modulation of 1/3), and the pulse period was 20 ms (corresponding to the pulse frequency of 50 Hz). Both CW-RF and PW-RF stimulation were generated intermittently (2 min field-on/8 min field-off) at an average input power of 1 W (30 dBm). The corresponding control groups (Ctrl) received no EMR signal. 

The RNA sequencing results showed that OLN-93 cells were almost not affected by CW-RF after both 6 and 48 h of EMR exposures, as there were no significantly different expressed genes (DEGs) detected. In contrast, there were several DEGs in both the 6-h and 48-h PW-RF groups, most of which had been up-regulated by EMR (Figure 1B). However, most of genes could not be well reproduced via RT-qPCR (Figure S1), except for C/EBPβ, the gene transcription of which was further shown by the integrative genomics viewer (IGV) (Figure 1C), and its transcription was effectively confirmed via multiple replications of RT-qPCR (Figure 1D). As shown in Figure 1D, the mRNA level of C/EBPβ was most significantly up-regulated after 6 h of PW-RF EMR, though with a slightly increased fold change (Ctrl = 1.002 ± 0.016, PW = 1.1223 ± 0.022, *p* < 0.001). It was also affected by 48 h of PW-RF EMR (Ctrl = 1.002 ± 0.015, PW = 1.1002 ± 0.033, *p* = 0.012), which was consistent with the RNA sequencing data. Nevertheless, 6 h of CW-RF EMR also increased the C/EBPβ mRNA level even though no difference was detected in the RNA sequencing data (Ctrl = 1.002 ± 0.016, PW = 1.066 ± 0.019, *p* = 0.016). No significant effect was detected in the C/EBPβ mRNA level in the 48-h CW-RF EMR group. 

In addition to oligodendrocytes, the CNS contains three other cell types, including neurons, microglia, and astrocytes. Neurons are the major component of the CNS and take responsibility for the processing and transmission of cellular signals. Microglia act as the first, and principle, form of active immune defense in the CNS. Astrocytes connect the entire CNS and perform many important and complex functions in the healthy system. Whether C/EBPβ in these cell types would also be affected by 2.4 GHz EMR is still unknown. We examined the mRNA level of C/EBPβ after radiation in the neuronal cell line HT-22 and found that the transcription of C/EBPβ was only up-regulated by 48-h CW-RF EMR but not by 6-h CW-RF EMR or PW-RF, which was distinct from OLN-93 (Figure 1E, Ctrl = 1.002 ± 0.020, RF = 1.177 ± 0.057, *p* = 0.010). Unlike OLN-93 and HT-22, the transcription of C/EBPβ in both the microglial cell line BV2 and in primary astrocytes was completely unaffected by both PW-RF and CW-RF EMR (Figure 1F,G). These findings indicated that 2.4 GHz EMR regulates C/EBPβ transcription differently depending on the cell type.

**Figure 2:**
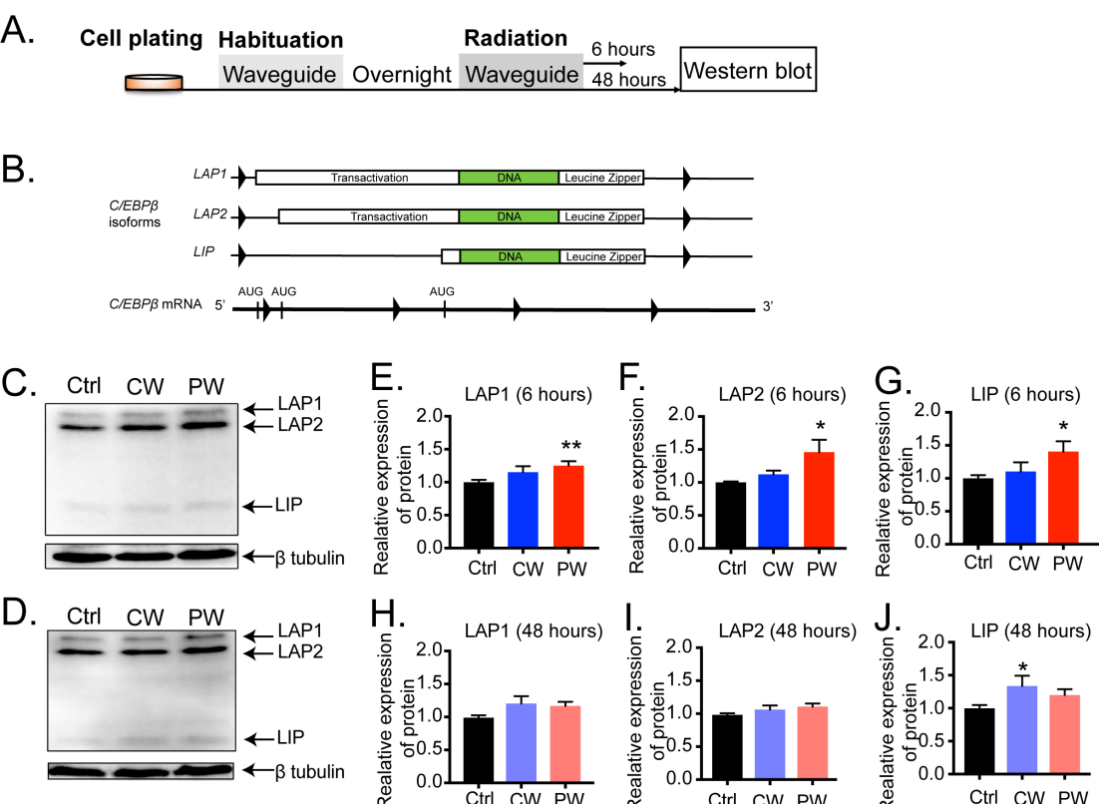
Expression of C/EBPβ was distinctly altered in oligodendroglial cells after 2.4 GHz EMR. (**A**) Experimental procedures. Cells were plated and habituated to the environment overnight, after which, they were dissected for analysis. (**B**) Three C/EBPβ isoforms were generated from a single exon mRNA via leaky ribosome scanning. (**C**,**D**) Western blotting of C/EBPβ isoforms after 6 h and 48 h of EMR exposure. (**E**–**G**) Relative expression of the three C/EBPβ isoforms was significantly enhanced after 6 h of PW-RF exposure but not after CW-RF exposure (n = 9–20 per group). (**H**–**J**) Forty-eight h of PW-RF exposure did not affect any of the C/EBPβ isoforms, while CW-RF exposure only increased LIP expression (n = 9–21 per group). * *p* < 0.05, ** *p* < 0.01 vs. Ctrl.

C/EBPβ mRNA yields three different protein isoforms mainly generated from a single exon gene via leaky ribosome scanning [10]. We next examined the protein expression levels of these isoforms after both 6 h and 48 h of 2.4 GHz EMR (Figure 2A). Figure 2B shows that the three isoforms of C/EBPβ were translated from a single C/EBPβ mRNA. Our results showed that 6 h of RF-EMR, particularly PW-RF EMR, significantly altered the expression levels of C/EBPβ isoforms but not CW-RF exposure (Figure S2 and Figure 2C–J). The expression of LAP1 (Figure 2E, F(2, 46) = 4.991, *p* = 0.011, one-way ANOVA), LAP2 (Figure 2F, F(2, 46) = 3.991, *p* = 0.027, one-way ANOVA), and LIP (Figure 2G, F(2, 46) = 3.562, *p* = 0.036, one-way ANOVA) significantly increased in the PW-RF group, although with a slightly increased fold change, but generally did not change in the CW-RF group, which was consistent with the mRNA level (Figure 1D). Nevertheless, 48 h of CW-RF EMR slightly up-regulated the expression of LIP (Figure 2J, F(2, 48) = 4.114, *p* = 0.022, one-way ANOVA). This effect did not occur with LAP1 or LAP2 (Figure 2H,I). These data indicated that short-term PW-RF exposure was more likely to disrupt the transcription and expression of C/EBPβ in oligodendroglial cells than CW-RF.

### 2.2. C/EBPβ-Interacting Proteins in Oligodendroglial Cells Were Altered by 2.4 GHz EMR Exposure

**Figure 3:**
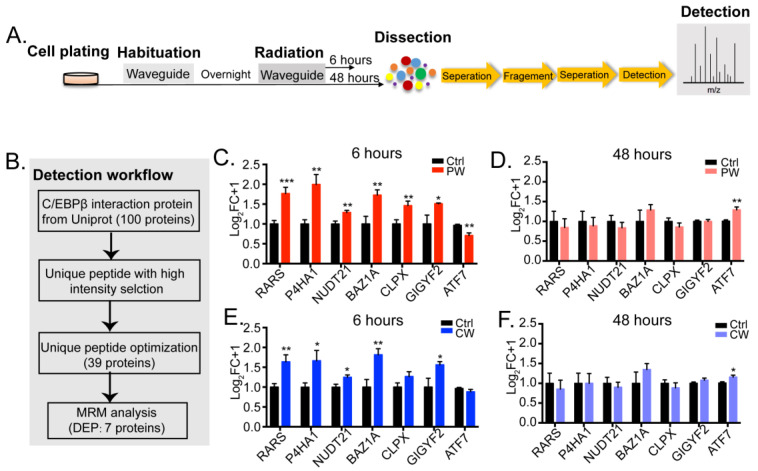
Expression of C/EBPβ-interacting proteins mostly altered after 6 h of exposure to 2.4 GHz EMR. (**A**) MRM experimental procedures. Cells were plated and habituated to the environment overnight, after which, the cell proteins were dissected to render peptides. Peptides were further fragmented, separated, and detected based on the *m*/*z* ratio. (**B**) Detection of MRM flow. (**C**,**E**) Six hours of PW-RF and CW-RF exposure up-regulated six interacting proteins, with the increase being more marked in the PW-RF group. ATF7 was significantly down-regulated by PW-RF but not CW-RF (n = 8–9 per group). (**D**,**F**) Only the expression of ATF7 was up-regulated by 48 h of EMR exposure (n = 8–9 per group). * *p* < 0.05, ** *p* < 0.01, *** *p* < 0.001 vs. Ctrl. DEP: differentially expressed protein.

C/EBPβ exerts its transcriptional activity by recruiting different proteins. We used multiple reaction monitoring (MRM) to examine the protein levels of C/EBPβ-interacting proteins after 6 and 48 h of 2.4 GHz EMR (Figure 3A). About 100 proteins from the Uniprot database that are predicted to interact with C/EBPβ were included (https://www.uniprot.org/uniprot/P21272#interaction, accessed on 10 May 2023). Before running the MRM procedure, Skyline was used to select proteins with high intensity and unique peptides from the spectral library, which left only 39 candidates for MRM analysis. The results showed that the expression levels of most of the candidates were not changed after 2.4 GHz EMR, as only seven differentially expressed proteins (DEPs) were detected (Figure 3B). 

It is notable that all seven candidates’ expressions were mostly altered by 6 h but not by 48 h of radiation, and particularly by PW-RF EMR (Figure 3C, RARS: Ctrl = 1.000 ± 0.089, PW = 1.767 ± 0.158, *p* = 0.001; P4HA1: Ctrl = 1.000 ± 0.105, PW = 1.996 ± 0.250, *p* = 0.004; NUDT21: Ctrl = 1.000 ± 0.073, PW = 1.290 ± 0.052, *p* = 0.007; BAZ1A: Ctrl = 1.000 ± 0.193, PW = 1.729 ± 0.130, *p* = 0.008; CLPX: Ctrl = 1.000 ± 0.105, PW = 1.463 ± 0.112, *p* = 0.009; GIGYF2: Ctrl = 1.000 ± 0.225, PW = 1.512 ± 0.017, *p* = 0.029; ATF7: Ctrl = 1.000 ± 0.027, PW = 0.744 ± 0.065, *p* = 0.002). Six-hour CW-RF also altered the expression of these candidates, but the effect was much weaker than that of PW-RF (Figure 3E, Supplementary Tables S2 and S3). 

Most of the DEPs were significantly up-regulated, consistent with the change in the expression of C/EBPβ. Forty-eight-hour radiation with both PW-RF and CW-RF had a very weak effect on most of the interacting proteins (Figure 3D, ATF7: Ctrl = 1.000 ± 0.033, PW = 1.292 ± 0.071, *p* = 0.003; Figure 3F, ATF7: Ctrl = 1.000 ± 0.033, PW = 1.157 ± 0.048, *p* = 0.024). The mRNA level of *Serpine1*, an encoding gene of another interacting protein of C/EBPβ, was also up-regulated by 6 h of PW-RF EMR but not by other radiations (Figure S3, Ctrl = 1.005 ± 0.027, PW = 1.102 ± 0.022, *p* = 0.009). These data indicated that 6-h exposure to PW-RF EMR not only increased the expression of C/EBPβ but also increased the expression of its potential interacting proteins, which may cooperate with C/EBPβ to exert its transcriptional activities.

### 2.3. Transcriptional Activity of C/EBPβ Was Enhanced after Exposure to 2.4 GHz EMR in Oligodendroglial Cells

**Figure 4:**
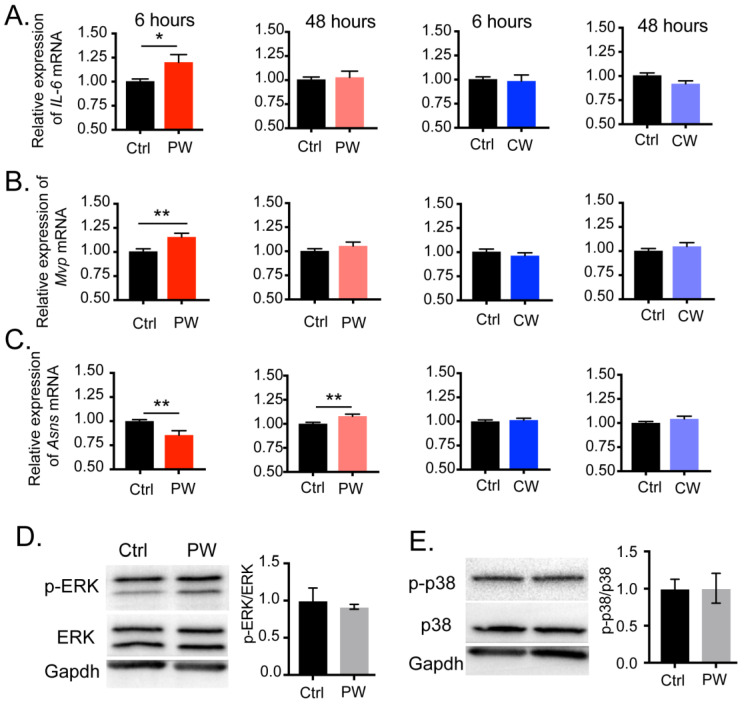
Transcription of C/EBPβ target genes was generally altered after 6 h of exposure to 2.4 GHz EMR, but no activation of upstream signaling was detected. (**A**,**B**) mRNA levels of both *Mvp* and *IL-6* were significantly enhanced after 6 h of exposure to PW-RF but not CW-RF, nor after 48 h of radiation from both (*Mvp*: n = 9; *IL-6*: n = 18 per group). (**C**) mRNA level of *Asns* was significantly down-regulated by 6 h of PW-RF but unaffected by 6 h of CW-RF exposure. However, it was up-regulated by 48 h of radiation from both, albeit with a rather slight fold change (n = 10–14 per group). (**D**) Western blot and the relative expression of p-ERK/ERK in oligodendroglial cells after 6 h of PW-RF exposure (n = 3). (**E**) Western blot and the relative expression of phos-p38/p38 in oligodendroglial cells after 6 h of PW-RF exposure (n = 3). * *p* < 0.05, ** *p* < 0.01 vs. Ctrl.

Next, we examined the transcriptional activity of C/EBPβ after exposure to 2.4 GHz EMR. We measured fourteen reported downstream genes of C/EBPβ in total, but only three were detected to be changed by 2.4 GHz EMR, with the remainders being unaffected by either types of EMR treatment (Supplementary Tables S4 and S5). C/EBPβ was first identified as a nuclear factor for Interleukin-6 (IL-6) by its specific binding to a response element in the *IL-6* genes [16]. Our results showed that 6 h of exposure to PW-RF significantly up-regulated the mRNA level of *IL-6* (Figure 4A, PW 6 h: Ctrl = 1.004 ± 0.023, RF = 1.201 ± 0.080, *p* = 0.027). Mvp has been reported to contain putative binding sites for C/EBPβ and may be involved in signal transduction [15]. As shown in Figure 4B, the mRNA level of *Mvp* was similar to that of *IL-6*; i.e., it was significantly increased by 6 h of exposure to PW-RF only (PW 6 h: Ctrl = 1.006 ± 0.028, RF = 1.156 ± 0.039, *p* = 0.006). *Asns* is an amino-acid-responsive gene, and the overexpression of individual isoforms of C/EBPβ (LAP1, LAP2, or LIP) could significantly inhibit the enhanced promoter activity of ASNS in histone-deprivation medium, suggesting that C/EBPβ has a role in preventing the excessive activation of ASNS [17]. Our results consistently showed the significant down-regulation of the mRNA level of *Asns* after 6 h of exposure to PW-RF (Figure 4C, PW 6 h: Ctrl = 1.000 ± 0.015, RF = 0.856 ± 0.046, *p* = 0.008). It is worth noting that the transcription level of *Asns* was up-regulated after 48 h of PW-RF exposure in our data, though with a very slightly increased fold change (Figure 4C, PW 48 h: Ctrl = 1.001 ± 0.014, RF = 1.079 ± 0.021, *p* = 0.007). Our results indicated that 6 h of exposure to PW-RF EMR specifically enhanced the transcription of downstream genes of C/EBPβ in oligodendroglial cells.

As a transcription factor, the expression and activation of C/EBPβ is regulated by a number of upstream signal cascades, including Erk, GSK β, and p38 [18,19]. We therefore considered the possibility that the changes in C/EBPβ expression and activity that we have observed above might be regulated by other signals responding directly to the RF-EMR. To explore this possibility, we specifically examined the activation of Erk and p38 signaling after 6 h of PW-RF EMR but found no significant activation (Figure S4 and Figure 4D,E, Ctrl = 1.000 ± 0.170, PW = 0.917 ± 0.033, *p* = 0.654 for pERK/ERK; Figure 4E, Ctrl = 1.000 ± 0.127, PW = 1.001 ± 0.201, *p* = 0.982 for p-p38/p38). More efforts with delicate strategies are needed in the future to search for the molecules that respond directly to RF-EMR. Our results indicated that 6 h of exposure to PW-RF EMR increased the expression and also specifically enhanced the transcriptional activity of C/EBPβ in oligodendroglial cells.

## 3. Discussion

Much epidemiological research has been carried out to elucidate the effect of RF EMR in sleep disorders [6], infertility [20], and tumors [21,22]. Most of the research has focused on tumors, particularly brain tumors, due to concerns regarding the frequent exposure of human brains to mobile phones, but despite this attention, the association between RF and tumors remains controversial. Early studies found no evident risk of brain tumors associated with cellular telephone use [23,24]. However, more recent studies have found that prolonged exposure to CDMA-modulated cell phone radiofrequency radiation promotes the incidence of malignant glioma in rats [21,25]. Our results showed that 2.4 GHz PW-RF EMR altered the expression and transcriptional activity of C/EBPβ in OLN-93 cells. As C/EBPβ is known to play an important role in glioblastoma [15], these findings highlight the possibility of a connection between exposure to 2.4 GHz EMR and the development of glioblastoma. According to Richter-Landsberg et al., OLN-93 is not yet differentiated in low serum conditions but expresses myelin sheath genes in high-serum-containing media [26]. Even though OLN-93 was cultivated in 10% FBS-containing medium in our tests, it may have some mature-oligodendrocyte-like characteristics, but it still does not exactly match mature oligodendrocytes in the brain. Furthermore, the in vivo system is probably more sophisticated than we realized, since mature oligodendrocytes in the brain create myelin sheaths around the axons and engage in complex interactions with neurons and other types of cells. Systemic energy absorption and interaction with electromagnetic fields in the body is quite different from what we observed in the in vitro system [27]. More concentrated efforts should therefore be made to identify and evaluate potential links between 2.4 GHz EMR exposure and the development of glioblastoma.

In addition to their possible role in correlations between RF-EMR and glioblastoma, the major function of oligodendrocytes in the brain is to produce myelin, and our finding that 2.4 GHz EMR exposure altered the expression of C/EBPβ and its interacting proteins in OLN-93 leads us to speculate that there may be a correlation between RF-EMR and myelination. Indeed, several of the related genes examined in our study have been previously reported to be associated with myelination-related functions. *Rars* encodes the cytoplasmic Arginine-tRNA ligase (ArgRS), a protein that catalyzes the attachment of amino acids to cognate tRNAs during protein synthesis. Clinical studies have reported that *Rars* variants impair ArgRS activity and cause classic hypomyelination presentation with nystagmus and spasticity, which indicates that *Rars* plays a role in myelination-related processes [28]. In a demyelination model used in the study of MOG-35-55-induced experimental autoimmune encephalomyelitis (EAE) [29,30], the crucial role of IL-6 in the induction phase of EAE was reported by Y. Okuda et al. [29], and the protective effect of IL-6 blockade against EAE via the inhibition of Th17 cells’ development was reported by Satoshi Serada et al. [30]; the results suggested that IL-6 may be involved in demyelination. Asparagine synthetase is encoded by *Ansn*, the mutation of which leads to asparagine synthetase deficiency. Clinical reports showed that *Ansn* mutation caused brain atrophy and delayed myelination [31,32]. In summary, our data suggested a possible association between RF-EMR and myelination functions. However, more extraordinary efforts should be conducted to assess this hypothesis.

RF EMR usually produces thermal effects on biological systems due to the energy absorption by the organism. In humans, exposure to high levels of RF energy may cause immediate health damage (tissue necrosis, headaches, etc.) because the body is not able to dissipate the excessive amounts of heat generated [1,33]. Besides its thermal effects, RF EMR also causes non-thermal effects when the energy is insufficient to cause significant heating [34]. In our study, we focused on the non-thermal effects of RF-EMR by restricting the input power to 1 W (30 dBm) and exposing cells in an intermittent way (2 min field-on/8 min field-off) to avoid the heating effects on cells. In addition, the SAR values of the samples in our study were within the range of 0.23–0.80 W/kg, which is well below the maximum limit of 2 W/kg recommended by the European guidelines for limiting exposures to RF EMR [35].

Signal modulation is widely used in wireless communication systems to enable signals to carry information efficiently. The possible differences in biological effects between continuous and modulated EMR have attracted the attention of a number of investigators over the years, but unfortunately, these results are rather controversial. According to a 2008 study, GSM-modulated RF-EMR significantly increased lipid peroxidation induced by tert-butyl hydroperoxide in SH-SY5Y cells but not continuous signals [36]. However, the same group in a 2009 study reported that continuous RF-EMR, but not a GSM-modulated signal, increased menadione-induced ROS levels in SH-SY5Y cells [37]. A 2011 review mentioned that amplitude-modulated RF EMR may have certain effects on the human CNS, although the effects reported were relatively minor [38]. Our data showed that PW-RF EMR, an amplitude-modulated signal form, disrupted the expression and activity of C/EBPβ in oligodendroglial cells more significantly than CW-RF (Figure 1, Figure 2, Figure 3 and Figure 4). However, there are still no known mechanisms to explain this difference.

Among the three isoforms of C/EBPβ, LAP1 and LAP2 contain both the transactivation and basic leucine-zipper domains, whereas LIP lacks the transactivation domain and forms non-functional heterodimers with LAP1 and LAP2. The ratio of LAP/LIP has a dramatic impact on transcriptional activation. A moderate increase in the LAP/LIP ratio resulted in significantly higher transcriptional activation of a CAT reporter gene with a high-affinity binding site for LAP in its promoter, according to an in vitro study [10]. However, this occurred in a simplified system in which exogenously expressed LAP and LIP almost exclusively bound to the promoter of the reporter gene. It is worth noting that within living cells, C/EBPβ isoforms interact with a variety of other transcription factors and other co-regulators in a diverse range of genes to alter transcriptional activity. For example, LIP homodimers interfere with the binding of Yin-Yang 1 (YY1), a transcriptional repressor protein, to the CXCR4 promoter, thus relieving YY1-induced transcriptional repression [32]. LAP1 has been shown to interact with cyclin D1 to promote β-casein expression and the differentiation of mammary epithelial cells [39]. Meanwhile, in the CXCR4 promoter, LAPs could not interact with the promoter despite the relatively high levels of endogenous LAPs [40]. Although the LAP/LIP ratio in our results was not affected by 6 h of PW-RF EMR, we still detected three targeted genes whose transcription levels were distinctly altered by PW-RF EMR. These results indicated that after PW-RF EMR, C/EBPβ isoforms in oligodendrocytes might interact with various co-regulators, in turn regulating the transcriptional activity of the C/EBPβ. In summary, our study revealed that 6 h of pulse-modulated RF-EMR has a significant effect on the expression and transcriptional activity of C/EBPβ in oligodendroglial cells.

## 4. Materials and Methods

### 4.1. Waveguide Exposure Apparatus

**Figure 5:**
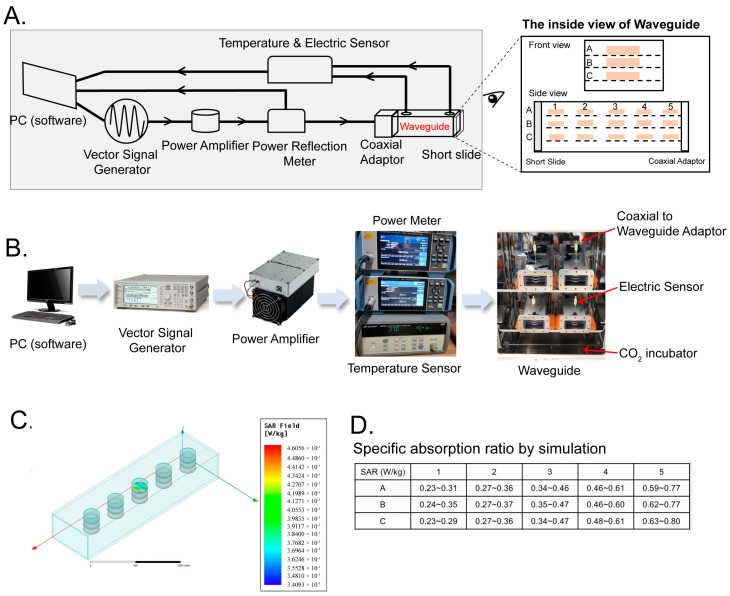
The waveguide exposure system. (**A**) Composition of EMR radiation system (**left**) and inside view of the waveguide (**right**). PMMA brackets were designed, each with five designated locations for 35 mm Petri dishes, and placed in three layers within the waveguides. The top, middle, and bottom layers were named layer A, layer B, and layer C, respectively, with the five locations in each layer designated as 1 to 5 (**right**). (**B**) Images of parts of the waveguide system. (**C**) **Left**: HFSS simulated spatial distribution and range of SAR within each dish in the waveguide. **Right**: The heatmap of the SAR value within a dish. (**D**) The SARs of culture media in all the Petri dishes.

We constructed an EMR exposure environment using waveguides, where the cells were exposed to uniform EMR stimulation with well-defined waveforms, avoiding the interference of the metallic structures of the cell incubator to the EMR distribution. As shown in Figure 5, a PC-controlled vector signal generator (N5181A, Agilent Technologies, Santa Clara, CA, USA) was used to generate predefined waveforms as input signals to the EMR exposure system. The signals were then amplified by a power amplifier (ZHL-30W-262-S+, Mini-Circuits, New York, NY, USA) before they were sent through a power reflection meter (NRT2, ROHDE&SCHWARZ, Munich, Germany) and then delivered into a rectangular waveguide. The power reflection meter measured EMR power in real time and fed this back to the PC software, which constantly adjusted the EMR power to maintain the preset level, and monitored it throughout the experiments. The temperature and the composition of the gases inside the waveguide were kept uniform with the environment in the cell incubator by using a pair of ventilators on each waveguide. The temperature inside the waveguide was measured using a temperature sensor, and temperature data (Supplementary Table S6) were collected using a data acquisition module (34901A, Keysight, Santa Rosa, CA, USA) coupled to a data collector (34972A, Keysight, Santa Rosa, CA, USA), which fed the data back to the PC software to allow us to record the temperature throughout the experiment.

On one end of the waveguide, the EMR signal from the output port of the power reflection meter was fed into the waveguide through a coaxial adaptor. The EMR signal propagated along the axial direction of the waveguide and was completely reflected by a short-circuit load (short slide) on the other end of the waveguide. The input EMR signals and the reflected EMR signals combined to form a standing wave. As a result, the input EMR signal was absorbed by the fifteen Petri dishes of cells or media within the waveguide. The residual reflected EMR passed out through the coaxial adaptor and was transmitted back to the power reflection meter, where it was recorded.

The EMR field distribution within the waveguide was simulated using HFSS, a Finite-Element Method tool. Specifically, we simulated the field distribution in the 2 mL culture medium in each of the fifteen positions in the system and their corresponding specific absorption ratios (SAR) (Figure 5C,D). The SAR value within each dish varied from 0.34 W/kg to 0.46 W/kg, with the largest variations occurring along the axial direction of the waveguide. The SAR of the culture medium in every Petri dish was within the range of 0.23~0.80 W/kg, with the higher SAR values occurring in Petri dishes closer to the coaxial adaptor. This variation was inevitable, since the EMR power was partially absorbed by the culture media along its propagation path, so its propagating power gradually attenuated with the distance along the propagating direction. Fortunately, the SAR values between the dishes within each stack were much more uniform, with the SAR variation between dishes A, B, and C in each stack being less than 10%. Taking advantage of this, we were able to minimize the influence of the variations in EMR strength along the waveguide axis by separating the Petri dishes into three groups, with each group containing one layer.

Even though we placed the control and RF waveguides in the same CO_2_ incubator and used a pair of ventilators to keep the gas and temperature constant in each waveguide, we were still concerned about unanticipated factors that might have had an impact on the effects that we observed. To obviate this, we switched the control and RF waveguides between each batch of experiments during the RT-qPCR and Western blotting tests, meaning that the left waveguide was set as a control and the right waveguide was the RF one for the first batch, the left waveguide was set as the RF and the right waveguide was a control one for the second batch, and so on. We believe this strategy improved the consistency of our results.

### 4.2. Cell Culture

The oligodendroglial cell line OLN-93 was kindly provided by Prof. Quanhong Ma from Soochow University with the permission of Prof. Zhi-Cheng Xiao of Monash University. OLN-93 was a permanent oligodendroglial cell line derived from primary rat brain glial cultures [26]. HT-22 (an immortalized mouse hippocampal cell line) and BV2 (a microglial cell line) were kindly provided by Prof. Piye Niu of Captical Medical University and Prof. Yanqing Wang of Fudan University, respectively. All cells were grown in DMEM with 10% fetal bovine serum (GIBCO) at 37 °C and 5% CO_2_ with 100% humidity. Cells were grown in 35 mm Petri dishes and seeded at ~1 million/dish for 6-h exposure experiments and ~0.3 million/dish for 48-h exposure experiments studies. In order to avoid acute stress effects, all cells were adapted to the waveguide apparatus by transferring them from the culture incubators to the waveguide ~12 h before the radiation experiments. All cells tested negative for mycoplasma before the experiments began.

### 4.3. Isolation and Culture of Primary Astrocytes

All animal experiments were approved by the Institutional Animal Care and Use Committee (IACUC) of Westlake University. Primary astrocyte isolation was performed according to a published protocol with minor modifications [41]. Two-day old rat pups were decapitated, and the skulls were removed. After the meninges were removed, the cerebral cortices were separated, sectioned, and digested using 50% DMEM containing trypsin for 15 min in an incubator at 37 °C and 5% CO_2_ while being gently swirled every 5 min. After stopping digestion with FBS containing DMEM, the tissue with suspension cells was collected in a tube and mechanically triturated with a 1 mL pipette to further separate the cells without creating bubbles. The resulting cell suspension was passed through a 50 µm nylon pouch, and then, the cells were plated in a poly-d-lysine-coated T 75 flask with 10% FBS and penicillin/streptomycin containing DMEM. They were fed every three days by replacing half of the medium with fresh medium. Ten days after plating, the flask was shaken at 250 rpm for 15–18 h in the incubator to remove the poorly attached microglia and oligodendrocytes. The purity of astrocytes was checked via the microscopic examination of their morphology. The primary astrocytes were then digested with 5 mM EDTA containing medium for 10 min while being shaken at 80 rpm in the incubator, and the suspension was then centrifuged at 1000 rpm for 5 min to obtain cell pellets. The cell pellets were resuspended with 10% FBS and penicillin/streptomycin containing DMEM, and the astrocytes were plated in poly-d-lysine-coated 35 mm dishes at a density of about ~0.5 million/dish. After overnight adhesion, the primary astrocytes were subjected to RF-EMR. During the experiments, the cells were fed by replacing half of the medium with fresh medium every three days.

### 4.4. RNA Extraction and Transcriptome Analysis

Total RNA was extracted immediately after appropriate EMR exposure timepoints using an ice-cold TRIZOL reagent (15596026, ThermoFisher, Waltham, MA, USA). Total cellular RNA was extracted according to the manufacturer’s instructions, and it was evaluated using a kaiaoK5500^®^Spectrophotometer (Kaiao, Beijing, China). The RNA Nano 6000 Assay Kit of the Bioanalyzer 2100 system (Agilent Technologies, Santa Clara, CA, USA) was used to evaluate RNA integrity numbers (RINs) and concentrations. Details about sequencing libraries’ construction and data analysis are introduced in the Supplementary Materials.

### 4.5. Cell Lysate Preparation and Western Blotting

Immediately after the termination of EMR exposure, cells were dissected in ice-cold RIPA (9806S, CST, Danvers, MA, USA) with fresh protease inhibitors (4693116001, Roche, Basel, Switzerland), incubated on ice for 10 min, and centrifuged at 12,000× *g* for 20 min at 4 °C. Supernatants of samples were assayed for protein content, and the protein concentration was measured using the BCA method (23227, ThermoFisher, Waltham, MA, USA) and then diluted to equalize a protein concentration of 1.5 μg/μL. A total of 30 µg protein per sample was used to load onto 12% SDS polyacrylamide gel and then transferred onto PVDF membrane. The membrane was blocked with 5% BSA in 0.02% Tween-20 tris-buffered saline (TBST) for 1 h at room temperature and probed with primary antibody (1:1000, anti-C/EBPβ (ab32358, Abcam, Cambridge, UK); 1:5000, anti-β-tubulin (10094-1-AP, Proteintech, Rosemont, IL, USA); 1:1000 anti-ERK (#4695, CST, Danvers, MA, USA); 1:1000 anti-p-ERK (#4370, CST, Danvers, MA, USA); 1:1000 anti-p38 (#9212S, CST, Danvers, MA, USA); 1:1000 anti-p-ERK (#9211S, CST, Danvers, MA, USA); anti-Gapdh (10494-1-AP, Proteintech, IL, USA) at 4 °C overnight and then incubated with HRP-conjugated anti-rabbit immunoglobulin G (IgG) (1:5000; A00098, GeneScript, Nanjing, China) for 1 h at room temperature. The membrane was rinsed in TBST and scanned in an Amersham Imager 680 (GE Healthcare System, Chicago, IL, USA) after incubation with a chemiluminescent detection reagent (RPN2232, Amersham, Arlington Heights, IL, USA). The immunoblots were analyzed with ImageJ to measure the optical density of the bands of C/EBPβ. The relative C/EBPβ expression was calculated by normalizing the intensity of C/EBPβ to that of β-tubulin.

### 4.6. Reverse Transcription and Real-Time Quantitative Polymerase Chain Reaction

The reverse transcription with random primers was conducted according to the Superscript First-Strand Synthesis System (RR037A, TAKARA, Beijing, China). Quantitative reverse transcription polymerase chain reaction (PCR) amplification of the complementary DNA was performed on samples in triplicate with Power SYBR Green PCR Master Mix (11201ES08*, YEASEN, Shanghai, China). The relative mRNA expression was normalized to the internal control Actb. The primers for examining C/EBPβ and targeted genes’ transcription levels are shown in Supplementary Table S1. The qPCR product of C/EBPβ was sequenced to ensure the specificity of primers and the accuracy of the transcriptional results.

### 4.7. Peptide Preparation

Cells were rinsed in ice-cold PBS three times and lysed using 50 µL of ice-cold lysis buffer (6 M urea and 2 M thiourea in 100 mM ammonium bicarbonate). Samples were transferred into a PCT-MicroTube (Pressure Biosciences Inc., Easton, MA, USA) and further subjected to protein digestion procedures using the Barocycler NEP2320-45K (Pressure Biosciences Inc., Easton, MA, USA) as previously described [42]. The PCT procedure for cell lysis was 90 cycles under 45,000 psi for 30 s and under ambient pressure for 10 s at 30 °C. After PCT lysis, proteins were reduced using 10 mM tris (2-carboxyethyl) phosphine (TCEP, #T4708, Sigma, St. Louis, MO, USA ) and alkylated using 40 mM iodoacetamide (IAA, Sigma, #SLCD4031) while being shaken at 800 rpm in the dark for 30 min. Reduced proteins were first digested using 2.5 μg of LysC (Hualishi Tech. Ltd., Beijing, China) in a PCT procedure, after which, 2 µg of trypsin (Hualishi Tech. Ltd., Beijing, China) was added to further digest the peptides using another PCT procedure. The digestion was then stopped by adjusting the pH to 2−3 using 1% trifluoroacetic acid (TFA). Peptides were next desalted using C18 SILICA Microspin columns (Nest Group Inc., ThermoFisher, Waltham, MA, USA) before MS analysis.

### 4.8. Multiple Reaction Monitoring (MRM) and Data Analysis

The digested peptides were injected into a nanoflow DIONEX UltiMate 3000 RSLCnano System coupled with a TSQ Altis Plus (Thermo Fisher Scientific™). For each sample, 500 ng of peptide was separated across a 30 min LC gradient (10–30% buffer B: 98% ACN, 0.1% FA; buffer A: 2% ACN, 0.1% FA) at a flowrate of 300 nL/min (trapcolumn, 3 μm, 100 Å, 20 mm × 75 μm i.d.; analytical column, 1.9 μm, 120 Å, 150 mm × 75 μm i.d.). The cycle time was 2 s, and the resolutions of Q1 and Q3 were 0.7 and 1.2, respectively. The retention time was predicted via iRT, and the isolation time window was 5 min. The data acquired via the MRM experiment were analyzed using Skyline [20147306]. The peak area of each peptide was calculated to their Log values, and the relative expressions of the RF groups were normalized to their control groups.

### 4.9. Statistical Analysis

Data consisted of mean ± standard error of mean (SEM) and were analyzed using SPSS 23. The different expressed genes (DEGs) analysis in RNA sequencing data was compared between radiation groups and their relative control groups by using DESeq2, and the criteria were FDR < 0.05, log_2_ fold change > 0.585, or log_2_ fold change < −0.585. The data for the mRNA expression of C/EBPβ and its target genes were tested with two-tailed Student’s t tests. The quantification of Western blotting was assessed using a one-way analysis of variance (ANOVA) with Bonferroni post hoc tests where appropriate. For MRM results, data were analyzed using two-tailed Student’s *t* tests. *p* < 0.05 was considered statistically significant.

## 5. Conclusions

The 2.4 GHz frequency range is widely used in modern electronic devices and is frequently experienced in our daily lives, but its impacts on gene expression and function in oligodendrocytes are unclear. Our data showed that exposure to pulse-modulated 2.4 GHz EMR for a period of 6 h has the potential to regulate the expression and function of the transcriptional factor C/EBPβ in oligodendroglial cells. The inference therefore cannot be discounted that exposure to RF-EMR may be having unanticipated biological effects.

## Data Availability

Data available on request due to restrictions, e.g., privacy or ethical.

## Supplementary Materials

The supporting information can be downloaded at: https://www.mdpi.com/article/10.3390/ijms241311131/s1.
