# Supplementary material for: Expression and Activity of the Transcription Factor CCAAT/Enhancer-Binding Protein β (C/EBPβ) Is Regulated by Specific Pulse-Modulated Radio Frequencies in Oligodendroglial Cells
Source: Int J Mol Sci. 2023 Jul 5;24(13):11131. doi: 10.3390/ijms241311131 (PMC10342410; doi:10.3390/ijms241311131)
Supplement: Supplementary file 1 [file ijms-24-11131-s001.zip › ijms-2418930-supplementary.pdf]

Supplementary Material

Expression and Activity of the Transcription Factor CCAAT/Enhancer-Binding Protein β (C/EBPβ) Is Regulated by Specific Pulse-Modulated Radio Frequencies in Oligodendroglial Cells

Bing Huang *, Weihao Zhao, Xue Cai, Yumin Zhu, Yingxian Lu, Junli Zhao, Nan Xiang, Xiaofei Wang, Hu Deng, Xiaping Tang, Lingyu Liu, Yanyu Zhao and Yigong Shi *

*** Correspondence:**

Bing Huang, bhuangfd@stu.edu.cn; Yigong Shi, syg@westlake.edu.cn

Supplementary Materials and Methods

1. The waveguide exposure apparatus

To keep the power density absorbed by cultured cells as consistent as possible throughout the EMR radiation processes, we designed to place the 35-mm petri dishes in five chosen positions along the axial direction of the waveguide, where the EMR field strength was maximal according to the numerical calculations. Petri dishes were held by a custom-made bracket of PMMA that fits the size of the cavity inside the waveguide. The distance between the center of each two chosen positions was 7 cm. At each position, a stack of three petri dishes was placed along the vertical central line of the corresponding cross-section, with the petri dishes vertically aligned to each other and the gap between two adjacent dishes was 1.5 cm. The top layer, middle layer, and bottom layer petri dishes are named as layer A, layer B, and layer C, respectively. As shown in Figure 1A, cells on layer A of each waveguide were stimulated for 6 hours,

while those at layer B were stimulated for 48 hours. The rest layer C were filled by petri dishes with 2 mL medium. During experiments, after dissecting cells exposed for 6 hours on layer A, petri dishes with 2 mL media were placed back to layer A to maintain the uniform of the EMR field and the EMR stimulation was kept on for another 42 hours to accomplish 48 hours exposure. During the entire EMR stimulation process, all the fifteen positions were filled with petri dishes with 2 mL culture medium either with or without cells so that the EMR distribution in the system is stable throughout the experiment. Each cell dish taken out during the stimulating time span will be replaced by new dishes with 2 mL culture medium.

To make sure the influence of possible thermal effect of the EMR signals, we also recorded the temperature variation of the culture medium in each petri dish with over-night continuous stimulation or without stimulation. The temperature of the control groups (no EMR stimulation) and the RF groups (with continuous 30 dBm EMR stimulation for ~12 hours) are listed in Supplementary Table S6. The difference between the two groups after 12 hours of incubation was 0~0.3°C, which was acceptable because the difference is comparable to the scale of the error of measurement.

2. RNA sequencing libraries construction and data analysis

Only samples with RIN > 7.0 were used to conduct library preparation and RNA sequencing process. A total amount of 2 μg RNA per sample was used as input material for the RNA sample preparations. Sequencing libraries were generated using NEBNext® UltraTM RNA Library Prep Kit for Illumina® (#E7530L, NEB, USA)

following the manufacturer's instructions. Concentration of library was measured using a Qubit® RNA Assay Kit in Qubit® 3.0 and then diluted to 1 ng/μl. The clustering of the index-coded samples was performed on a cBot cluster generation system using HiSeq PE Cluster Kit v4-cBot-HS (Illumina) according to the manufacturer's instructions. After cluster generation, the libraries were sequenced on an Illumina platform and 150 bp paired-end reads were generated. The processed sequencing data was downloaded from the company and obtained the QC metrics using FastQC with default parameters. rRNA reads were removed by mapping the processed data to rRNA sequence in Rat using bowtie with parameters "-v 1 -M 1 -m 10000 --best". Unmapped reads were used for further analysis. Then STAR was used to map the sequence to the Rat genome Rnor_6.0 with default parameters. After mapping, we obtained the read count by featureCount using the following parameters: -T 2 -s 0 -p -t exon -g gene_id. Then edgeR was used to identify differentially expressed genes.

Supplementary Figures and Tables

1. Supplementary Figures

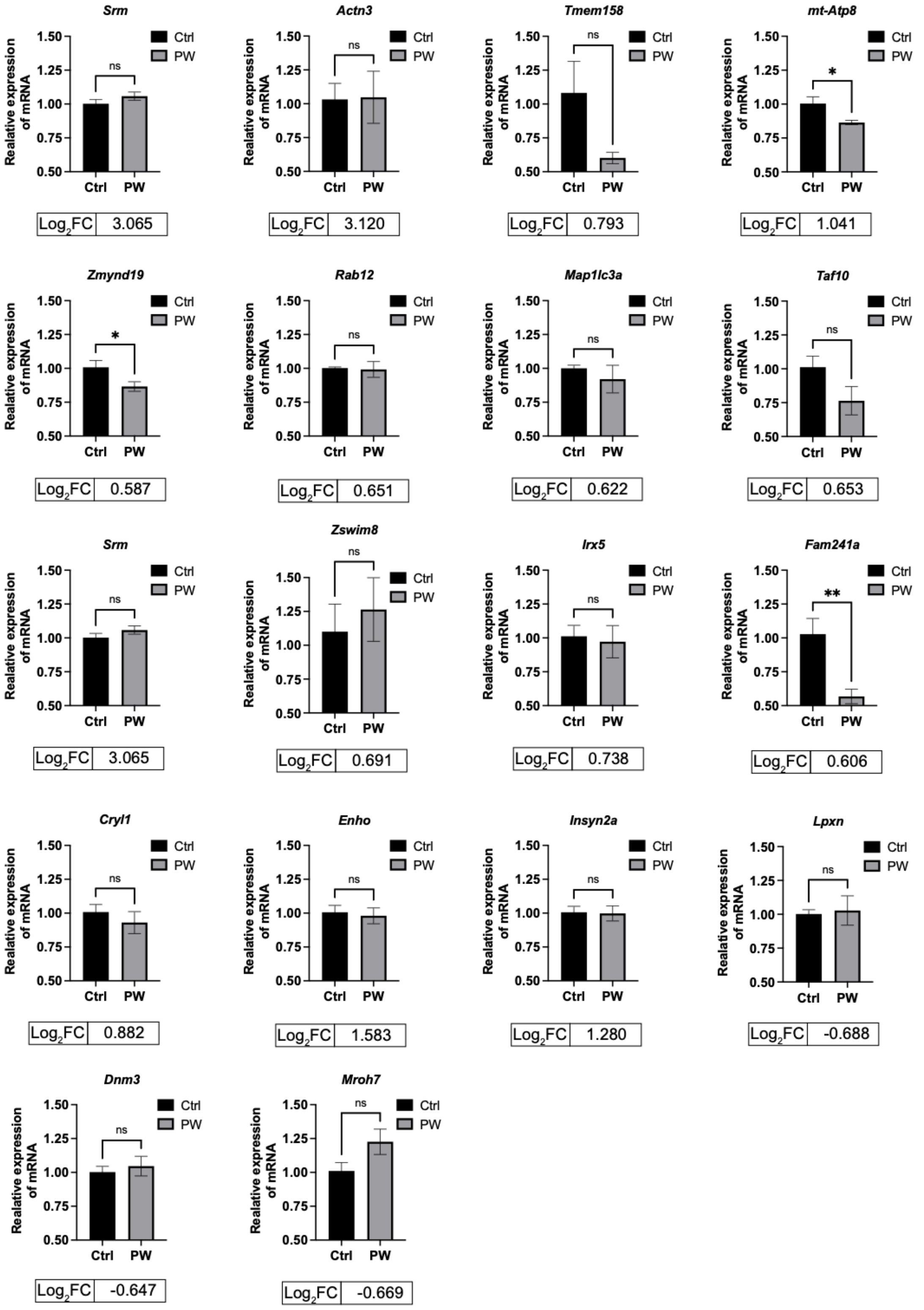

Supplementary Figure S1. Transcription levels of other DEGs except for C/EBPβ shown in Figure 1B. mRNA levels of those genes detected significantly changed by RNA sequencing were examined in independently generated samples after 6 hours of exposure to PW-RF. The RT-qPCR results failed to replicate the RNA sequencing data (Figure 1B). The table below each graph showed the $\log_2$ fold change in RNA sequencing results.

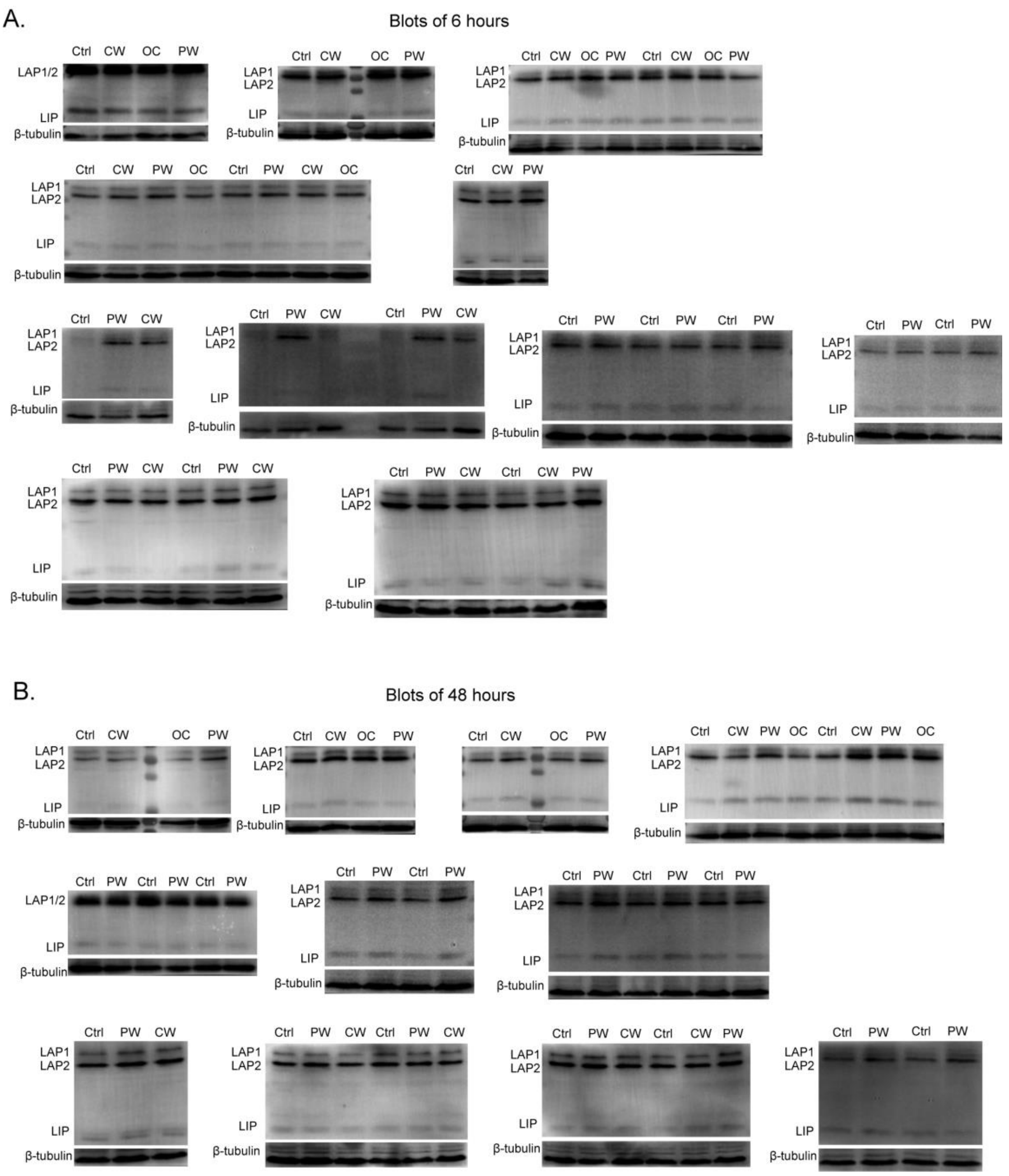

Supplementary Figure S2. Western blots of C/EBPβ shown in Figure 2C-J. (A,B) All the blots of C/EBPβ isoforms in both 6-hour and 48-hour groups.

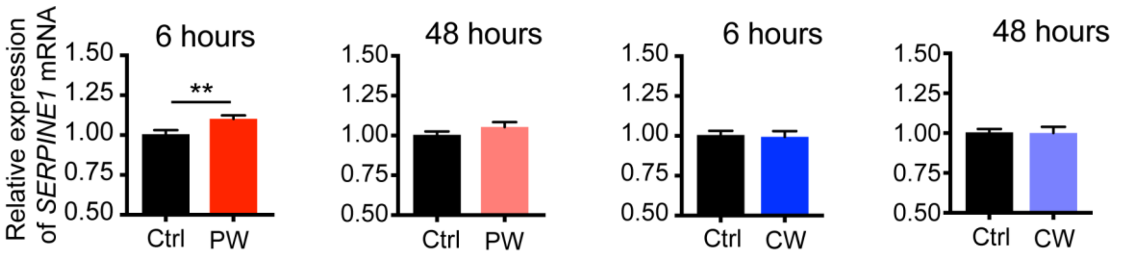

Supplementary Figure S3. Transcription of *Serpine1* was mostly altered after 6 hours of exposure to PW-RF EMR. mRNA level of *Serpine1* was enhanced by 6 hours of PW-RF exposure but not by CW-RF or both 48 hours RF-EMR (n=10-14 per group). $^{**}p < 0.01$ vs. Ctrl.

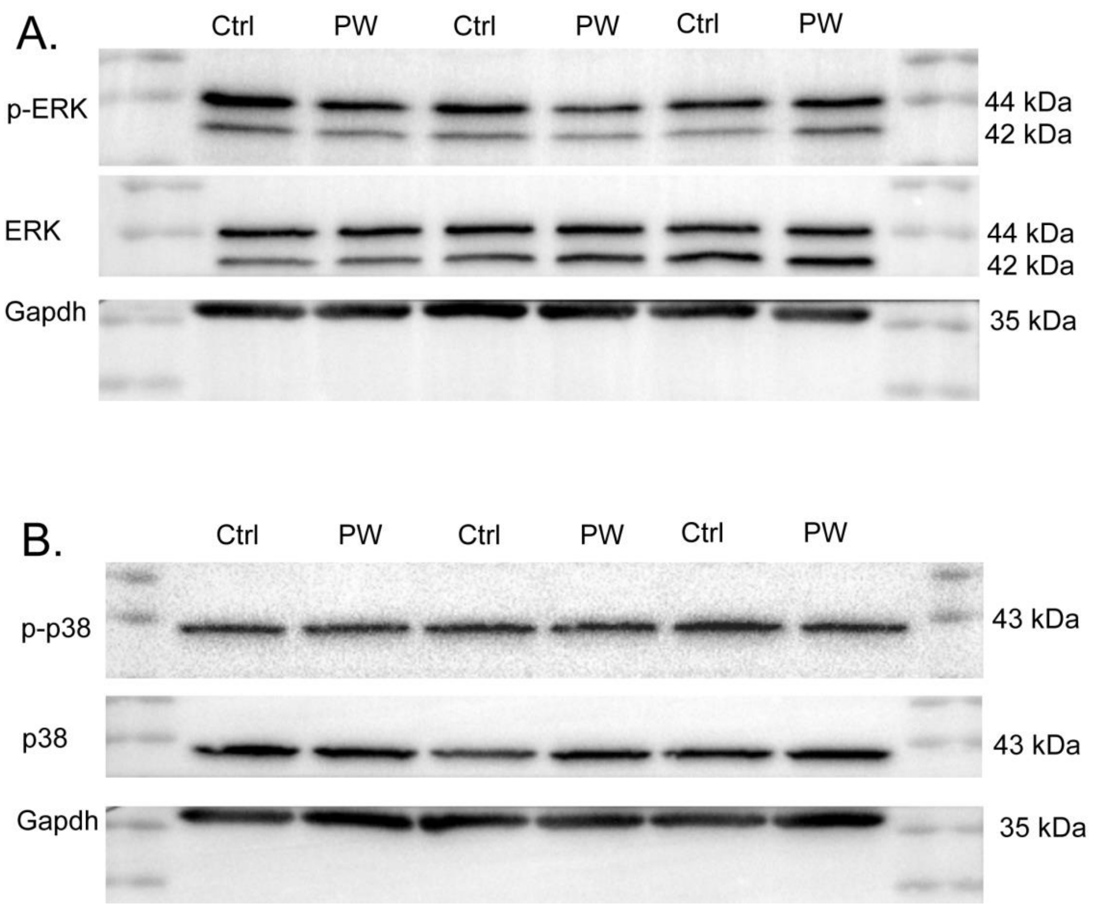

Supplementary Figure S4. Western blots of ERK and p38 signaling shown in Figure 4D-E. (A-B) Blottings of p-ERK/ERK and phos-p38/p38 in oligodendroglial cells after 6 hours of exposure to PW-RF.

2. Supplementary Tables

Supplementary Table S1. The designed primers for examining C/EBPβ and targeted genes transcription level.

Gene Name	Forward Primer (5′-3′)	Reverse Primer (5′-3′)
R_C/EBPβ	ATCGACTTCAGCCCCTA	CTCACGTAACCGTAGTC
R_Actb	GGTCATCACTATCGGCAAT	GTGTTGGCATAGAGGTCTT
R_Mvp	AGTTCAAGGAGATGACAGAG	TCGGTGATGAGAGTGGAT
R_IL6	AATCTGCTCTGGTCTTCTG	ACTCCTTCTGTGACTCTAAC
R_Asns	TCGGAAGAACACAGACAG	GGCAGAGACAGGTAATAGG
R_Cavin1	AACACCGTGAGCAAGTTG	CCTCGTTGACCTCCAGTT
R_Ctgf	GGTCTCTTCTGCGACTTC	AACTGCTTTGGAAGGACTC
R_Shc1	CGTGGAGGTCTTACAGTC	GTGAGAGTGATCGGCATT
R_Klhdc4	ATGGGCAAGAAGGGAAAG	GGAACCGAACAGAAGAGG

R_Cxcl14	GGTCCAAGTGTAAGTGTTC	ATCTTCTCCTCGCAGTGT
R_Mmp15	AGCCTACACCTACTTCTACA	GAACCACCTCCTCCATCT
R_Kdma5	TCCACCACTTCCTGATGT	CTTACTAGCCGCCAGAAC
R_Eif4g	GTCTCCTCACCACGATTG	CAAGCACATCCTGTAGCA
R_Vegfa	CTTGTTCAGAGCGGAGAA	CCTTGGCTTGTCACATCT
R_Vegfb	CAACACCAAGTCCGAATG	CTTCACAGCACTCTCCTT
R_Alkbh5	AGGATGAGTGCTCCAAGA	GCTGGTAGTCGTTGATGA
M_ C/EBPβ	AGCGACGAGTACAAGATG	CTGCTCCACCTTCTTCTG
M_Actb	GCACCACACCTTCTACAA	TACGACCAGAGGCATACA

Supplementary Table S2. Descriptions for abbreviations of C/EBPβ interacting proteins.

**Protein Name**	**Description**
RARS	Arginine-tRNA ligase (UniProtKB: P40329)
P4HA1	Prolyl 4-hydroxylase subunit alpha-1 (UniProtKB: P54001)
NUDT21	Cleavage and polyadenylation specificity factor subunit 5 (UniProtKB: Q4KM65)
BAZ1A	Bromodomain adjacent to zinc finger domain, 1A (UniProtKB: F1M4U9)
CLPX	ATP-dependent Clp protease ATP-binding subunit clpX-like, mitochondrial (UniProtKB: Q5U2U0)
GIGYF2	GRB10 interacting GYF protein 2 (UniProtKB: A0A096MJI4)
ATF7	Atf7 protein (UniProtKB: B0BMY0)

Supplementary Table S3. The relative expression levels of C/EBPβ interacting proteins after 6 hours of exposure to CW-RF EMR.

**Gene Name**	**Relative Expression in Control Group**	**Relative Expression in CW-RF Group**	***p* Value**
RARS	1.000 ± 0.089	1.638 ± 0.176	0.007
P4HA1	1.000 ± 0.105	1.666 ± 0.258	0.037
NUDT21	1.000 ± 0.073	1.250 ± 0.057	0.016
BAZ1A	1.000 ± 0.193	1.816 ± 0.150	0.004
CLPX	1.000 ± 0.105	1.268 ± 0.122	0.121
GIGYF2	1.000 ± 0.225	1.564 ± 0.078	0.043
ATF7	1.000 ± 0.027	0.916 ± 0.062	0.233

Supplementary Table S4. Descriptions for abbreviations of C/EBPβ target genes.

Gene Name	Description
IL-6	Interleukin 6 (Gene ID: 24498)
Mvp	Major vault protein (Gene ID: 64681)
Asns	Asparagine synthetase (Gene ID: 25612)
Cavin1	Caveolae associated protein 1 (Gene ID: 287710)
Ctgf	Cellular communication network factor 2 (Gene ID: 64032)
Shc1	SHC adaptor protein 1 (Gene ID: 85385)
Klhdc4	Kelch domain containing 4 (Gene ID: 307917)
Cxcl14	C-X-C motif chemokine ligand 14 (Gene ID: 306748)
Mmp15	Matrix metallopeptidase 15 (Gene ID: 291848)
Kdm5a	Lysine demethylase 5A (Gene ID: 312678)
Eif4g	Eukaryotic translation initiation factor 4, gamma 2 (Gene ID: 361628)
Vegfa	Vascular endothelial growth factor A (Gene ID: 83785)
Vegfβ	Vascular endothelial growth factor B (Gene ID: 89811)
Alkbh5	alkB homolog 5, RNA demethylase (Gene ID: 303193)

Supplementary Table S5. Transcription level of target genes of C/EBPβ. mRNA levels of target genes of C/EBPβ. mRNA expression of most of genes were not affected by any radiations (n=14-19 per group for 6 h groups; n=10-20 per group for 48 h groups). F.C represents Fold change.

Gene	6 hours				48 hours			
	PW		CW		PW		CW	
	F.C	*p* value	F.C	*p* value	F.C	*p* value	F.C	*p* value
Cavin1	1.03	0.12	1.03	0.20	0.98	0.34	0.93	0.01
Ctgf	1.05	0.27	1.02	0.65	1.05	0.03	1.07	0.09
Shc1	1.04	0.07	1.04	0.19	1.04	0.06	1.03	0.16
Klhdc4	0.97	0.62	1.02	0.87	1.03	0.79	1.05	0.45
Cxcl14	0.99	0.78	1.03	0.44	1.05	0.22	0.98	0.47
Mmp15	1.02	0.64	1.04	0.21	1.00	0.92	1.02	0.39
Kdm5a	1.03	0.46	1.03	0.51	1.04	0.50	0.97	0.52
Eif4g	0.96	0.19	0.98	0.71	1.05	0.39	0.98	0.21
Vegfa	1.00	0.78	1.04	0.31	1.13	0.04	1.05	0.99
Vegfβ	0.89	0.35	1.02	0.31	1.05	0.18	1.00	0.53
Alkbh5	0.95	0.18	1.06	0.06	1.00	0.98	0.99	0.37

Supplementary Table S6. The temperature of cell culture medium after overnight exposure to RF-EMR. Petri dishes with 2 mL culture medium were placed in the waveguide and stimulated by continuous 30 dBm EMR stimulation (RF) or without stimulation (Control) overnight, after which temperature of medium were measured by a thermistor immediately. Number 1-5 represents designated locations of petri dishes from the side of short slide to the side of coaxial adaptor.

**Temperature (℃)**	**1**	**2**	**3**	**4**	**5**
**Control**	35.6	35.3	35.3	35.3	35.3
**RF**	35.8	35.6	35.3	35.4	35.4
